# Bladder Pain Syndrome/Interstitial Cystitis Is Associated with Hyperthyroidism

**DOI:** 10.1371/journal.pone.0072284

**Published:** 2013-08-21

**Authors:** Shiu-Dong Chung, Shih-Ping Liu, Ching-Chun Lin, Hsien-Chang Li, Herng-Ching Lin

**Affiliations:** 1 Division of Urology, Department of Surgery, Far Eastern Memorial Hospital, Banciao, Taipei, Taiwan; 2 Sleep Research Center, Taipei Medical University Hospital, Taipei, Taiwan; 3 Department of Urology, National Taiwan University Hospital and College of Medicine, Taipei, Taiwan; 4 Graduate Institute of Biomedical Informatics, College of Medical Science and Technology, Taipei Medical University, Taipei, Taiwan; 5 School of Health Care Administration, Taipei Medical University, Taipei, Taiwan; 6 School of Medical Laboratory Sciences and Biotechnology, Taipei Medical University, Taipei, Taiwan; Northwestern University, United States of America

## Abstract

**Background:**

Although the etiology of bladder pain syndrome/interstitial cystitis (BPS/IC) is still unclear, a common theme with BPS/IC patients is comorbid disorders which are related to the autonomic nervous system that connects the nervous system to end-organs. Nevertheless, no study to date has reported the association between hyperthyroidism and BPS/IC. In this study, we examined the association of IC/BPS with having previously been diagnosed with hyperthyroidism in Taiwan.

**Design:**

Data in this study were retrieved from the Longitudinal Health Insurance Database. Our study consisted of 736 female cases with BPS/IC and 2208 randomly selected female controls. We performed a conditional logistic regression to calculate the odds ratio (OR) for having previously been diagnosed with hyperthyroidism between cases and controls.

**Results:**

Of the 2944 sampled subjects, there was a significant difference in the prevalence of prior hyperthyroidism between cases and controls (3.3% vs. 1.5%, *p*<0.001). The conditional logistic regression analysis revealed that compared to controls, the OR for prior hyperthyroidism among cases was 2.16 (95% confidence interval (CI): 1.27∼3.66). Furthermore, the OR for prior hyperthyroidism among cases was 2.01 (95% CI: 1.15∼3.53) compared to controls after adjusting for diabetes, coronary heart disease, obesity, hyperlipidemia, chronic pelvic pain, irritable bowel syndrome, fibromyalgia, chronic fatigue syndrome, depression, panic disorder, migraines, sicca syndrome, allergies, endometriosis, and asthma.

**Conclusions:**

Our study results indicated an association between hyperthyroidism and BPS/IC. We suggest that clinicians treating female subjects with hyperthyroidism be alert to urinary complaints in this population.

## Introduction

Hyperthyroidism is one of the most common endocrine disorders, defined as an excess production of triiodothyronine (T3) hormone and an increase in peripheral thyroxine (T4) [1], [2]. Hyperthyroidism is nearly 10-times more common in women than men, and its incidence increases with advancing age [3], [4]. The hyperthyroidism status can enhance the cellular response to adrenergic activity, causing an increase in sympathetic activity and a decrease in parasympathetic activity [5]. Various clinical presentations and nosologic subtypes with different etiologies can cause overt and subclinical hyperthyroidism [6], [7].

It is well documented in the literature that features of hyperthyroidism, such as depression and anxiety, may be similar to those observed in patients with psychiatric disease [8], [9]. Evidence also suggests that psychological disturbances, bladder problems, and other pain disorders may share a common etiology [10], [11]. Moreover, Watkins et al. found morbidity and a poor quality of life (QOL) associated with patients who had interstitial cystitis/bladder pain syndrome (BPS/IC) and depressive or anxiety disorders [12].

IC/BPS is a chronic pain syndrome of unknown etiology, characterized by pain perceived to originate from the bladder, urinary urgency and frequency, and nocturia [13]. It primarily affects women and has a marked effect on their QOL [14], [15]. The prevalence of IC/BPS is 0.45%∼12.6% depending upon methodological factors, such as the index or scale used for the diagnosis [16], [17]. Although the etiology of IC/BPS is still unclear, a common theme with BPS/IC patients is comorbid disorders that may be related to the autonomic nervous system which connects the nervous system to end-organs [18].

Nevertheless, no study to date has reported the association between hyperthyroidism and BPS/IC. In order to both fill this gap in the literature and add to knowledge surrounding the association of IC/BPS with hyperthyroidism, we examined the association between IC/BPS and having previously been diagnosed with hyperthyroidism in Taiwanese women.

## Methods

### Database

Data used in this case-control investigation were retrieved from the Longitudinal Health Insurance Database (LHID2000). The LHID2000, which was derived from the Taiwan National Health Insurance (NHI) program (with about a 98.5% coverage rate since its inception in 1995), includes all original medical claims for one million selected enrollees. These one million enrollees were randomly retrieved from all enrollees listed in the 2000 Registry of Beneficiaries (*n* = 23.72 million) under the Taiwan NHI program. The LHID2000 provides researchers an opportunity to trace all use of medical services for these 10^6^ enrollees since initiation of the NHI program in 1995. The Taiwan National Health Research Institute and some researchers have validated the representativeness of the LHID2000 relative to the entire population of NHI enrollees [19], [20]. Numerous studies employing the LHID2000 were published in internationally peer-reviewed journals [21].

This study was exempt from full review by the Institutional Review Board of Taipei Medical University because the LHID2000 consists of de-identified secondary data released to the public for research purposes.

### Study Sample

For cases in this case-control study, we first identified 1102 female subjects aged ≥18 years who had received their first-time diagnosis of BPS/IC (ICD-9-CM code 595.1 (chronic interstitial cystitis)) in an ambulatory care visit (including outpatient departments of hospitals and clinics) from January 1, 2001 to December 31, 2011. In order to increase the diagnosis validity of BPS/IC, this study only included cases who had ever received Cystistat® (sodium hyaluronate) treatment (*n* = 736). Under the Taiwan NHI program, prescriptions for Cystistat® are exclusively reserved for subjects diagnosed with BPS/IC. The NHI Bureau implements routine, sample cross-checks of each hospital’s claims with medical charts, followed by punitive measures for coding infractions, which deters diagnosis upcoding. Therefore, it is almost impossible that anyone without a clear diagnosis of BPS/IC would be eligible for this treatment. For cases, we assigned their first date of receiving a diagnosis of BPS/IC as the index date.

As to the selection of controls, we likewise retrieved subjects from the remaining enrollees in the registry of beneficiaries of the LHID2000. We excluded subjects who had ever received a diagnosis of BPS/IC since initiation of the NHI program in 1995. In total, 2208 female subjects (three for every subject with BPS/IC) were randomly selected to match the cases by age group (18∼29, 30∼39, 40∼49, 50∼59, 60∼69 and >69 years), monthly income, geographic region (northern, central, eastern, and southern Taiwan), and index year. For cases, the year of the index date was the year in which the subjects received their first diagnosis of IC/PBS. However, for controls, the year of the index date was simply a matched year in which subjects had visited a physician. We indicated the date of their first visit of a physician occurring during that matched year as the index date for controls.

### Exposure Assessment

We included cases with hyperthyroidism based on the principal diagnosis of ICD-9-CM code hyperthyroidism (ICD-9-CM code 242, thyrotoxicosis with or without goiter). In Taiwan, hyperthyroidism is generally diagnosed by specialists in endocrinology. In this study, we only selected cases with hyperthyroidism who had received two or more hyperthyroidism diagnoses prior to the index date, with at least one being made by a specialist in endocrinology.

### Statistical Analysis

The SAS statistical package (SAS System for Windows, vers. 8.2, Cary, NC, USA) was used to perform all statistical analyses. We used Pearson ^χ2^ tests to compare differences between cases and controls in terms of selected comorbidities (diabetes, coronary heart disease (CHD), obesity, hyperlipidemia, chronic pelvic pain (CPP), irritable bowel syndrome (IBS), fibromyalgia, chronic fatigue syndrome (CFS), depression, panic disorder, migraines, sicca syndrome, allergies, endometriosis, and asthma). We selected these comorbidities because of their potential associations with IC/PBS based on prior studies [22], [23]. We further performed a conditional logistic regression (conditioned on age group, monthly income, geographical location, and index year) to calculate the odds ratio (OR) for having been previously diagnosed with hyperthyroidism between cases and controls. The conventional *p*≤0.05 was used to assess statistical significance.

## Results

Table 1 shows the distributions of sociodemographic characteristics and comorbidities between cases and controls. After matching for age group, monthly income, geographic region, and index year, subjects with BPS/IC had higher prevalences of the comorbidities of CHD (*p* = 0.004), hyperlipidemia (*p* = 0.043), CPP (*p*<0.001), IBS (*p*<0.001), fibromyalgia (*p*<0.001), depression (*p*<0.001), migraines (*p*<0.001), sicca syndrome (*p* = 0.014), allergies (*p*<0.001), and asthma (*p*<0.001) than controls.

**Table 1:** Demographic characteristics of patients with bladder pain syndrome/interstitial cystitis (BPS/IC) and controls in Taiwan in 2001∼2011 (n = 2944).

Variable	Patients with BPS/IC (*n* = 736)		Controls (*n* = 2208)			*p* value	
	Total no.	Percent (%)	Total no.	Percent (%)			
Urbanization level						0.634	
1 (highest)	261	35.5	747		33.8		
2	208	28.3	618		28.0		
3	95	12.9	320		14.5		
4	102	13.8	286		13.0		
5 (lowest)	70	9.5	237		10.7		
Diabetes	115	15.6	309	14.0		0.275	
Coronary heart disease	115	15.6	254	11.5		0.004	
Obesity	10	1.4	21	1.0		0.348	
Hyperlipidemia	160	21.7	405	18.3		0.043	
Chronic pelvic pain	242	32.9	395	17.9		<0.001	
Irritable bowel syndrome	93	12.6	119	5.4		<0.001	
Fibromyalgia	256	34.8	526	23.8		<0.001	
Chronic fatigue syndrome	10	1.4	15	0.7		0.082	
Depression	89	12.1	143	6.5		<0.001	
Panic disorder	7	1.0	12	0.5		0.232	
Migraines	56	76	84	3.8		<0.001	
Sicca syndrome	16	2.2	22	1.0		0.014	
Allergies	26	3.5	32	1.5		<0.001	
Endometriosis	23	3.1	50	2.3		0.194	
Asthma	79	10.7	149	6.8		<0.001	

Table 2 presents the prevalences of prior hyperthyroidism between cases and controls. Of the 2944 sampled subjects, 58 (2.0%) had received a hyperthyroidism diagnosis before the index date. There was a significant difference in the prevalence of prior hyperthyroidism between cases and controls (3.3% vs. 1.5%, *p*<0.001). A conditional logistic regression analysis (conditioned on age group, monthly income, geographic location, and index year) revealed that compared to controls, the OR for prior hyperthyroidism among cases was 2.16 (95% confidence interval (CI): 1.27∼3.66). Furthermore, the OR for prior hyperthyroidism among cases was 2.01 (95% CI: 1.15∼3.53) than controls after adjusting for diabetes, CHD, obesity, hyperlipidemia, CPP, IBS, fibromyalgia, CFS, depression, panic disorder, migraines, sicca syndrome, allergies, endometriosis, and asthma.

**Table 2:** Prevalence, and crude and adjusted odds ratios (ORs) for prior hyperthyroidism among the sampled patients.

Presence of prior hyperthyroidism	Total (*n* = 2944)		Patients with bladder pain syndrome/interstitial cystitis (*n* = 736)		Controls (*n* = 2208)	
	*n*, Percent 9%)		*n*, Percent (%)		*n*, Percent (%)	
Yes	58	2.0	24	3.3	34	1.5
No	2,886	98.0	712	96.7	2,174	98.5
Crude OR (95% CI)	–		2.16** (1.27–3.66)		1.00	
Adjusted OR (95% CI)^a^	–		2.01* (1.15–3.53)		1.00	

CI, confidence interval; ORs were calculated by a conditional logistic regression which was conditioned on age, monthly income, and geographic region group.

* *p*<0.05;

** *p*<0.01.

a Adjusted for patient’s urbanization level, diabetes, coronary heart disease, obesity, hyperlipidemia, chronic pelvic pain, irritable bowel syndrome, fibromyalgia, chronic fatigue syndrome, depression, panic disorder, migraines, sicca syndrome, allergies, endometriosis, and asthma.

Table 3 displays the OR for prior hyperthyroidism among the sampled subjects according to age group. Among subjects aged ≥60 years, the OR for prior hyperthyroidism among cases was as high as 2.59 (95% CI: 1.01∼6.79) compared to controls after adjusting for diabetes, CHD, obesity, hyperlipidemia, CPP, IBS, fibromyalgia, CFS, depression, panic disorder, migraines, sicca syndrome, allergies, endometriosis, and asthma. However, among subjects aged 18∼39 years, there was no increased adjusted OR for prior hyperthyroidism among cases compared to controls.

**Table 3:** Odds ratios (ORs) for hyperthyroidism among patients with bladder pain syndrome/interstitial cystitis (BPS/IC) and controls, by age group.

Presence of hyperthyroidism	Age group (years)					
	18∼39		40∼59		≥60	
	Patients with BPS/IC *n*, Percent (%)	Controls *n*, Percent (%)	Patients with BPS/IC *n*,Percent (%)	Controls *n*,Percent (%)	Patients with BPS/IC *n*,Percent (%)	Controls *n*, Percent (%)
Yes	4 (2.1)	10 (1.6)	12 (3.6)	15 (1.5)	8 (3.8)	9 (1.5)
Crude OR^a^ (95% CI)	1.32 (0.41∼4.25)	1.00	2.54* (1.16∼5.56)	1.00	2.79* (1.04∼7.49)	1.00
Adjusted OR^b^ (95% CI)	1.21 (0.37∼3.96)	1.00	2.41* (1.11∼5.19)	1.00	2.59* (1.01∼6.79)	1.00

a CI, confidence interval; ORs were calculated by a conditional logistic regression which was conditioned on gender, monthly income, and geographic region group.

* *p*<0.05.

b Adjustments were made for patient’s urbanization level, diabetes, coronary heart disease, obesity, hyperlipidemia, chronic pelvic pain, irritable bowel syndrome, fibromyalgia, chronic fatigue syndrome, depression, panic disorder, migraines, sicca syndrome, allergies, endometriosis, and asthma.

## Discussion

To the best of our knowledge, this is the first large-scale population-based study that investigated the relationship between BPS/IC and hyperthyroidism. Our results demonstrated that patients with BPS/IC were 2.16-times more likely than controls to have had a previous diagnosis of hyperthyroidism. Even after adjusting for comorbid conditions, BPS/IC subjects were still at 2.01-times greater risk than comparison subjects for prior hyperthyroidism. We also independently investigated the associated between BPS/IC and hyperthyroidism of each age group. We found that among subjects aged ≥60 years, the OR for prior hyperthyroidism among cases was as high as 2.59 (95% CI: 1.01∼6.79) compared to controls after adjusting for comorbid conditions.

While there are no data available indicating any direct causality between BPS/IC and hyperthyroidism, we speculated that the association detected in this study may be due to shared risk. Previous studies reported that hyperthyroidism and anxiety have overlapping features [8], [24], [25]. A study by Kathol and Delahunt, found that anxiety in approximately half of patients with newly diagnosed and untreated hyperthyroidism [25]. That brings to mind how the concurrent presence of somatic thyroid symptoms artificially inflates levels of depression and anxiety. Rodewig stated that psychological symptoms in hyperthyroidism were similar to neurotic anxiety symptomatology and anxious depressive syndrome [26]. Furthermore, this raises the issue of concomitant BPS/IC symptoms and psychological symptoms possibly being widely prevalent. One review by Von Korff and Simon suggested that pain is associated with anxiety disorders and that anxiety symptoms such as worry and disturbed sleep are common among pain patients [27].

Moreover, changes in fluid excretion in hyperthyroidism and BPS/IC may serve as a mechanism for urinary frequency. Evered et al. suggested that urinary frequency in hyperthyroid patients may be a consequence of polyuria [28]. Hyperthyroidism is also associated with hyperdynamic circulation, which results in increased renal blood flow and an increased incidence of polyuria [29]. Moreover, the International Continence Society identifies BPS/IC as a complaint of suprapubic pain related to bladder filling, accompanied by other symptoms such as increased daytime and nighttime frequency, in the absence of proven urinary infection or other obvious pathology [30]. A prospective study of 15 patients showed that the mean daytime urinary frequency was 20 (range 7∼30) times, and after treatment, the average frequency of daytime urination was cut approximately in half [31]. The symptom of urinary frequency might be a shared risk between hyperthyroidism and BPS/IC. Furthermore, an imbalance of the automatic nervous system might be another pathogenic factor. Although the mechanism is still unclear, Chelimsky et al. suggested that patients with IC/BPS had comorbid autonomic nervous system disorders [18], and Ho et al. also indicated that the symptoms and signs of hyperthyroidism are thought to result from an imbalance of the automatic nervous system [32].

The principal strength of our study lies in its longitudinal and large population database, which enabled us to trace all cases of hyperthyroidism and IC/BPS during the study period and avoided problems of selection biases inherent in studies utilizing data taken from voluntary registries or hospital-referred study patients. Nevertheless, this study needs to be seen in the light of some limitations. First, hyperthyroidism and BPS/IC diagnoses from the database were reported by physicians and hospitals through ICD-9-CM codes, and may thus be less accurate than diagnoses made according to standardized criteria. However, we only included IC/BPS patients who had ever received Cystistat®, which is exclusively reserved for subjects diagnosed with BPS/IC. Furthermore, the NHI Bureau uses punitive measures for coding infractions to deter diagnosis upcoding. Second, we have limited information about the clinical characteristics of the women with BPS/IC used in this study, and were therefore unable to differentiate study participants according to the severity of their BPS/IC and were unable to evaluate whether subjects with more-severe BPS/IC had a higher risk of prior hyperthyroidism than those with mild BPS/IC. Last, this study might have suffered from some degree of a surveillance bias. As subjects with hyperthyroidism are more likely to have more-frequent checkups than healthy controls, it is possible that they were more likely to be diagnosed with BPS/IC purely on account of their increased exposure to the medical community. But, as BPS/IC is accompanied by both pain and increased urgency, and is only diagnosed in the absence of a urinary infection or other pathology, it is unlikely that subjects without hyperthyroidism would have been less likely to seek medical care.

Despite these limitations, our study revealed that there was an association between hyperthyroidism and BPS/IC after adjusting for comorbid conditions. We suggest that clinicians treating female subjects with hyperthyroidism be alert to urinary complaints in this population. In addition, the specific mechanisms which underlie this relationship are still unknown. Further study is needed to confirm our findings and explore the underlying pathomechanisms.

## References

[1] Wu EL, Chien IC, Lin CH, Chou YJ, Chou P (2013) Increased risk of hypothyroidism and hyperthyroidism in patients with major depressive disorder: A population-based study. J Psychosom Res 74: 233–237. 23438714 10.1016/j.jpsychores.2012.12.016

[2] Bielecka-Dabrowa A, Mikhailidis DP, Rysz J, Banach M (2009) The mechanisms of atrial fibrillation in hyperthyroidism. Thyroid Res 2: 4. 19341475 10.1186/1756-6614-2-4 PMC2680813

[3] Vanderpump MP (2011) The epidemiology of thyroid disease. Br Med Bull 99: 39–51. 21893493 10.1093/bmb/ldr030

[4] Vadiveloo T, Donnan PT, Cochrane L, Leese GP (2011) The Thyroid Epidemiology, Audit, and Research Study (TEARS): morbidity in patients with endogenous subclinical hyperthyroidism. J Clin Endocrinol Metab 96: 1344–1351. 21346066 10.1210/jc.2010-2693

[5] Chung SD, Chen YK, Chen YH, Lin HC (2011) Hyperthyroidism and female urinary incontinence: a population-based cohort study. Clin Endocrinol 75: 704–708. 10.1111/j.1365-2265.2011.04126.x 21623855

[6] Carlé A, Pedersen IB, Knudsen N, Perrild H, Ovesen L, et al (2011) Epidemiology of subtypes of hyperthyroidism in Denmark: a population-based study. Eur J Endocrinol 164: 801–809. 21357288 10.1530/EJE-10-1155

[7] Cooper DS (2003) Hyperthyroidism. Lancet 362: 459–468. 12927435 10.1016/S0140-6736(03)14073-1

[8] Demet MM, Ozmen B, Deveci A (2002) Depression and anxiety in hyperthyroidism. Arch Med Res 33: 552–556. 12505101 10.1016/s0188-4409(02)00410-1

[9] Hendrick VC, Garrick TR (2000) Endocrine and metabolic disorders. B.J Sadock, V.A Sadock (Eds.), Comprehensive textbook of psychiatry (7th ed), Williams & Wilkins, Philadelphia, PA, USA, 1808–1818.

[10] Weissman MM, Gross R, Fyer A, Heiman GA, Gameroff MJ, et al (2004) Interstitial cystitis and panic disorder: a potential genetic syndrome. Arch Gen Psychiatry 61: 273–279. 14993115 10.1001/archpsyc.61.3.273

[11] Weissman MM, Fyer AJ, Haghighi F, Heiman G, Deng Z, et al (2000) Potential panic disorder syndrome: clinical and genetic linkage evidence. Am J Med Genet 96: 24–35. 10686548 10.1002/(sici)1096-8628(20000207)96:1<24::aid-ajmg7>3.0.co;2-e

[12] Watkins KE, Eberhart N, Hilton L, Suttorp MJ, Hepner KA, et al (2011) Depressive disorders and panic attacks in women with bladder pain syndrome/interstitial cystitis: a population-based sample. Gen Hosp Psychiatry 33: 143–149. 21596207 10.1016/j.genhosppsych.2011.01.004 PMC3099040

[13] Clemens JQ, Meenan RT, Rosetti MC, Gao SY, Calhoun EA (2005) Prevalence and incidence of interstitial cystitis in a managed care population. J Urol 173: 98–102. 15592041 10.1097/01.ju.0000146114.53828.82

[14] Michael YL, Kawachi I, Stampfer MJ, Colditz GA, Curhan GC (2000) Quality of life among women with interstitial cystitis. J Urol 164: 423–427. 10893601

[15] Nickel JC, Payne CK, Forrest J, Parsons CL, Wan GJ, et al (2009) The relationship among symptoms, sleep disturbances and quality of life in patients with interstitial cystitis. J Urol 181: 2555–2561. 19375108 10.1016/j.juro.2009.02.030

[16] Leppilahti M, Tammela TL, Huhtala H, Auvinen A (2002) Prevalence of symptoms related to Interstitial Cystitis in women: a population based study in Finland. J Urol 168: 139–143. 12050508

[17] Rosenberg MT, Hazzard M (2005) Prevalence of interstitial cystitis symptoms in women: a population based study in the primary care office. J Urol 174: 2231–2234. 16280776 10.1097/01.ju.0000181203.82693.95

[18] Chelimsky G, Heller E, Buffington CA, Rackley R, Zhang D, et al. (2012) Co-morbidities of interstitial cystitis. Front Neurosci 6, 114. 10.3389/fnins.2012.00114 PMC3415690 22907988

[19] Cheng CL, Kao YH, Lin SJ, Lee CH, Lai ML (2011) Validation of the National Health Insurance Research Database with ischemic stroke cases in Taiwan. Pharmacoepidemiol Drug Safety 20: 236–242. 10.1002/pds.2087 21351304

[20] Kang JH, Chen YH, Lin HC (2010) Comorbidity profiles among patients with ankylosing spondylitis: a nationwide population-based study. Ann Rheum Dis 69: 1165–1168. 20375121 10.1136/ard.2009.116178

[21] Chen YC, Yeh HY, Wu JC (2011) Taiwan’s National Health Insurance Database: administrative health care database as study object in bibiometrics. Scientometrics 86: 365–380.

[22] Keller JJ, Liu SP, Lin HC (2012) A case-control study on the association between rheumatoid arthritis and bladder pain syndrome/interstitial cystitis. Neurourol Urodyn (in press). 10.1002/nau.22348 23129416

[23] Kang JH, Keller JJ, Chen YK, Lin HC (2012) Reflux esophagitis increased the risk of bladder pain syndrome/interstitial cystitis: A 3-year follow-up study. Neurourol Urodyn. (in press). 10.1002/nau.22270 22674619

[24] Trzepacz PT, McCue M, Klein I, Greenhouse J, Levey GS (1988) Psychiatric and neuropsychological response to propranolol in Graves’ disease. Biol Psychiatry 23: 678–688. 3370265 10.1016/0006-3223(88)90051-0

[25] Kathol RG, Delahunt JW (1986) The relationship of anxiety and depression to symptoms of hyperthyroidism using operational criteria. Gen Hosp Psychiatry 8: 23–28. 3943712 10.1016/0163-8343(86)90060-5

[26] Rodewig K (1993) Psychosomatic aspects of hyperthyroidism with special reference to Basedow’s disease. An overview. Psychother Psychosom Med Psychol 43: 271–277. 8378518

[27] Von Korff M, Simon G (1996) The relationship between pain and depression. Br J Psychiatry Suppl 30: 101–108. 8864155

[28] Evered DC, Hayter CJ, Surveyor I (1972) Primary polydipsia in thyrotoxicosis. Metabolism 21: 393–404. 4111512 10.1016/0026-0495(72)90052-2

[29] Vargas F, Atucha NM, Sabio JM, Quesada T, García-Estañ J (1994) Pressure-diuresis-natriuresis response in hyperthyroid and hypothyroid rats. Clin Sci 87: 323–328. 7955909 10.1042/cs0870323

[30] Abrams P, Cardozo L, Fall M, Griffiths D, Rosier P, et al (2002) The standardisation of terminology of lower urinary tract function: report from the Standardisation Sub-committee of the International Continence Society. Am J Obstet Gynecol 187: 116–126. 12114899 10.1067/mob.2002.125704

[31] Maher CF, Carey MP, Dwyer PL, Schluter PL (2001) Percutaneous sacral nerve root neuromodulation for intractable interstitial cystitis. J Urol 165: 884–886. 11176493

[32] Ho CH, Chang TC, Guo YJ, Chen SC, Yu HJ, et al (2011) Lower urinary tract symptoms and urinary flow rates in female patients with hyperthyroidism. Urology 77: 50–54. 21195824 10.1016/j.urology.2010.07.479

